# Determing an “autoimmune” phenotype in systemic JIA

**DOI:** 10.1186/1546-0096-9-S1-P150

**Published:** 2011-09-14

**Authors:** N Fischer, B Huegle, JP Haas

**Affiliations:** 1 Deutsches Zentrum für Kinder- und Jugendrheumatologie, Garmisch-Partenkirchen, Germany

## Background

Data from research, clinics and response to treatment point out to an autoinflammatory pathogenesis in Systemic onset Juvenile Idiopathic Arthritis (SoJIA). However a subgroup of patients present with **c**hronic **a**ctive course of disease, destructive **p**olyarthritis and production of **a**utoantibodies (CAPA-subtype) not responsive to anti IL-1 treatment.

## Aim

Determine phenotypic characteristics of the CAPA-subtype of SoJIA.

## Methods

Retrospective analysis in a cohort of 35 SoJIA patients well characterized according clinical course and ANA, Rheumatic factor and ANCA.

## Results

Autoantibodies have been found in 25,7% of the patients, with ANA being the most frequent auto-AK (table 1). There was a significant difference according the age of onset. Moreover females and carditis have been observed more frequently in the AK-pos. group.

**Table:** 

SoJIA-Patients	35			
**Observation (month)**	**mean 69** (range 1-262)			

	**n**	**%**	**n**	**%**

**Autoantibodies**	**pos**		**neg**	

	9	25,7	26	74,3
ANA	6	17,1		
RF	2	5,7		
ANCA	3	8,6		
	percentage for each group			
male	3	33,3	11	42,3
female	6	66,6	15	57,7
onset age (years)	6,03		3,54	
relapses	4		3,6	
joints at onset	2,1		3,1	
joints when AK pos	7			
MAS	1		1	
Carditis	6	66,7	13	50
SAA	2 of 4		8 of 12	
Amyloidosis	1		1	

## Conclusion

There is evidence for an autoimmune course of disease in SoJIA in a subset of patients with chronic polyarthritis but without relapses of autoinflammation. These patients probably will need a switch in treatment to prevent further joint destruction.

